# Dynamic prognostic model for kidney renal clear cell carcinoma (KIRC) patients by combining clinical and genetic information

**DOI:** 10.1038/s41598-018-35981-5

**Published:** 2018-12-04

**Authors:** Huiling Zhao, Yuting Cao, Yue Wang, Liya Zhang, Chen Chen, Yaoyan Wang, Xiaofan Lu, Shengjie Liu, Fangrong Yan

**Affiliations:** Research Center of Biostatistics and Computational Pharmacy, China pharmaceutical University, Nanjing, 210009 P. R. China

## Abstract

We aim to construct more accurate prognostic model for KIRC patients by combining the clinical and genetic information and monitor the disease progression in dynamically updated manner. By obtaining cross-validated prognostic indices from clinical and genetic model, we combine the two sources information into the Super learner model, and then introduce the time-varying effect into the combined model using the landmark method for real-time dynamic prediction. The Super learner model has better prognostic performance since it can not only employ the preferable clinical prognostic model constructed by oneself or reported in the current literature, but also incorporate genome level information to strengthen effectiveness. Apart from this, four representative patients’ mortality curves are drawn in the dynamically updated manner based on the Super learner model. It is found that effectively reducing the two prognostic indices value through suitable treatments might achieve the purpose of controlling the mortality of patients. Combining clinical and genetic information in the Super learner model would enhance the prognostic performance and yield more accurate results for dynamic predictions. Doctors could give patients more personalized treatment with dynamically updated monitoring of disease status, as well as some candidate prognostic factors for future research.

## Introduction

In the area of precision medicine, increasingly more attention is paid to the dynamic prognosis of disease status and progression. That is, according to the historical information and the dynamic information obtained after each follow-up, it would be relatively easy to predict future survival status and the time to the next failure event, which would alert the doctor early enough to take effective measures. Cancer prognosis and survival predictions are critical to physicians and patients in many aspects, such as monitoring the disease progression and planning early prevention and treatment, thus improving the quality of life of patients.

Kidney renal clear cell carcinoma (KIRC) is the eighth most common type of cancer, and it accounts for 70–80% of renal cell carcinoma. It is a kind of tumour with a relatively low degree of malignancy and slow development, and generally, no early clinical symptoms are revealed until the tumour volume is large enough to be found^1^. Although the early diagnosis is related with a high cure rate, the confirmed patients are mostly in the medium or late stages, when the mortality and recurrence rates are quite high. Therefore, KIRC is still a threat to human life and health as a malignant disease. It is necessary to conduct real-time information tracking, dynamic prognosis analysis for KIRC patients.

Currently, most of the prognostic analysis systems for renal cell carcinoma are based on clinical indicators but ignore the biological characteristics during the disease progression. University of California, Los Angeles (UCLA) scholars have established a UISS scoring system to predict the overall survival rate of patients with localized and metastatic renal cell carcinoma^2^. According to the pathological variables after renal cancer surgery, the Mayo Clinic has established the SSIGN scoring system based on the pathological stage, size, grade and necrosis of renal cancer, and predicts the survival and metastasis of patients through the system^3^. These two prognostic systems based on clinical information are widely used. However, the micro-environment often develops in the process of cancer development, which leads to significant differences in the expression of some genes. Among them, some contain important information about the patient’s condition and survival and provide important support for prognosis and dynamic predictions. Therefore, considering both clinical and genetic information can enhance the accuracy of prognostic systems and the related dynamic predictions by borrowing information from external features and internal mechanisms, as well as provide studies with potential biomarkers and therapeutic targets.

In this article, we set up a clinical model and a genetic model based on the Cox proportional hazards model to obtain the two sources information. By combining the clinical and genetic information represented by the cross-validated prognostic indices, we establish the Super learner model, which is a joint evaluation prognostic model. The combined model could be a more comprehensive reflection of the patients’ effective information, thus enhancing the predictive performance. Then, the most commonly used landmark method is introduced into the Super learner model for dynamic prediction. Through the prediction graph, doctors could have an overall view of the progression of the disease, conduct interventions at dangerous times, or replace the treatment in a timely manner to improve the relevance and effectiveness of the treatment, which is also a necessary condition for future precision medicine.

The rest of the article is organized as follows. In section 2, we briefly introduce the Super learner model and the dynamic prediction method. In section 3, we apply the proposed method to the KIRC dataset in the GDAC firehouse database, and the related results are presented. Section 4 is the summary of the entire article.

## Material and Methods

### Data collection

In this paper, we download the most recent KIRC patients’ genetic data (gdac.broadinstitute.org_KIRC.mRNAseq_Preprocess.Level_3.2016012800.0.0) and clinical data (gdac.broadinstitute.org_KIRC.Merge_Clinical.Level_1.2016012800.0.0) from the Broad GDAC firehouse ftp (http://gdac.broadinstitute.org/runs/stddata_2016_01_28/data/KIRC/20160128/) as the original data for the following analysis.

### Clinical and genetic model

In this paper, we first use the logarithmic rank test and Kaplan-Meier (KM) estimation to eliminate the clinical indicators that had no significant differences on the survival between the cancer group and the control group. Then, the Cox regression model is fitted for “backward screening” multivariate analysis, which is called the second screening^4,5^. After the two-step screening, the remaining clinical indicators are significantly related to KIRC patients’ survival time. Refitting the Cox model (Eq. (A.1) in the Supplementary Materials) with the selected indicators and compare the model performance with UISS model and SSIGN model, we finally determine the clinical model.

In regard to genetic data, the general survival analysis method (Cox proportional hazards model) has difficulty in analysing microarray data. The biggest problem is that the number of variables is much larger than the number of samples. That is, p ≫ n. To solve the problem, the LASSO method is introduced into the Cox model^6–8^ to reduce the dimensions according to Eq. (A.2) in the Supplementary Materials. Based on this penalty, the genetic model is established to screen the significant genes. These selected genes are associated with the survival time, having the possibility to become biomarkers or prognostic factors for KIRC patients.

### Super learner model

Cross-validated prognostic indices *CVPI*_*clin*_ and *CVPI*_*gene*_^6,9^ are determined from the clinical and genetic model, which represent the two sources information, and then are used to fit the Super learner model^10–12^. Details can be found in the Supplementary Materials. Through this approach, the patients’ clinical and genetic information is combined into a single prognostic model, and the following dynamic prediction is performed on it.

### Landmark dynamic prediction

To some extent, the patients’ data is dynamically updated, so the prognostic model should be constantly updated based on the latest data in order to avoid inaccuracies. To achieve this goal, based on the Super learner model, we construct a single “super prediction dataset”. By applying the landmark ipl* integral partial logarithmic likelihood model to the constructed dataset, the accurate dynamic prediction of a patient’s future survival rate can be realized^9,13^. Details can be found in the Supplementary Materials.

## Results

### Data pre-processing

From the Broad GDAC firehouse website, we download KIRC raw data.

Clinical raw data has 2837 clinical indicators and 537 samples. According to previous literature research^2,3,14,15^ and data cleaning up in different follow-up stages, we obtain 14 clinical indicators which are known related with KIRC survival and progression. The clinical indicators (in addition to survival time and status) are summarized in the Supplementary Materials Table A.1.

Genetic raw data has 20502 genes and 537 samples. Using the R package “DESeq2”, 5837 genes that are differentially expressed in tumour samples and control samples are screened out at a fold ratio = 2 and P-adjusted value = 0.05. All the screened out genes in the DESeq2 normalized expression matrix are fitted into the Cox proportional hazards model for univariate regression, 1432 genes with regression P value < 0.05 are finally selected. Then, the processed data matrix is logarithmically normalized to reduce the errors in the LASSO screening.

After the pre-processing, clinical data are matched with the genetic data. Finally, 127 matched tumour samples are obtained for the following analysis.

### Variable selection and single source model construction

For the clinical data, the 12 pre-treated clinical indicators (except for survival time and status) are analysed by the KM curve and univariate logarithmic rank test. In this way, the clinical indicators related to the survival time of KIRC patients are initially screened out.

Table A.2, Figs A.1 and A.2 (Supplementary Materials) indicate that there are nine clinical indicators having a significant impact on the survival rate of KIRC patients, which are the tumour grade, tumour laterality, tnm_m, tnm_n, tnm_t, tumour size, UISS score, necrosis and SSIGN score. It can also be concluded that the last three indicators nuclei_percent, diag_age and gender produce no significant effect. These three indicators are discarded, thus moving away from the next step.

Apart from UISS score and SSIGN score which are used to construct models for comparison, the 7 remaining significant clinical indicators selected in Table A.2 (Supplementary Materials) enter the second screening step for multivariate analysis. Through the Cox stepwise regression, 4 clinical indicators are screened out (Table 1). The regression coefficients are all above zero, thus indicating their positive relationships with the risk function, or in other words, with the risk of the patients’ death.

**Table 1:** Clinical indicators screened from Cox stepwise regression.

Clinical variable	Coefficient	P value	HR
Tnm_m_m1	1.121	0.002***	3.067
Grade_3	0.262	0.576	1.300
Grade_4	0.906	0.059	2.474
Tumour size	0.563	0.119	1.755
necrosis	0.654	0.052	1.923

^(*)^p < 0.05, ^(**)^<0.01, ^(***)^<0.001.

Based on the above analysis, we construct the candidate clinical models (Eq. (A.1) in the Supplementary Materials) with the 4 selected clinical indicators, the UISS score and the SSIGN score as covariates separately. The internal content of the exponential function of the model is defined as *PI*_*clin*_, and then, the corresponding values *CVPI*_*clin*_ are generated by leave one out cross validation. From Table 2, it can be concluded that the AUC of the model with SSIGN score as covariates is larger than the others. Interestingly, SSIGN score can be calculated from tumour metastasis, tumour size, tumour grade and necrosis status, which are just the 4 clinical indicators we selected above. Thus, we choose SSIGN score to fit the clinical model.

**Table 2:** Model performance comparison of candidate clinical models.

Candidate model	Covariate	AUC
1	4 selected indicators	0.775
2	SSIGN score	0.791
3	UISS score	0.662

**Figure 1:**
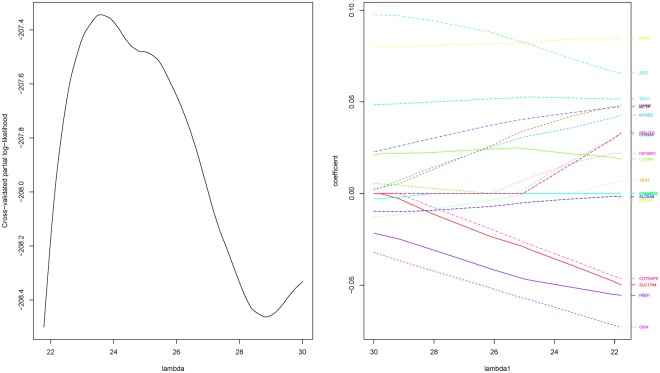
Selecting the optimal adjustment parameter by cross validation (left) and the LASSO screening process (right).

For genetic data, the LASSO-penalized Cox regression model (genetic model) is established. The cross-validation method is used to select the adjustment parameter *λ*. Figure 1 shows the optimal adjustment parameter and the screening process for the LASSO method under 500-fold cross validation. When the optimal adjustment parameter is 23.61, the number of genes screened out from the LASSO process is changed to 15, and the parameters corresponding to these significant variables are shown in the Supplementary Materials Table A.3.

Clearly, a total of 15 genes are selected by the genetic model. Among them, the regression coefficients of INHBE, IGFN1, L1CAM, etc. are greater than zero. This indicates that the greater the gene expression value is, the greater the risk ratio and the lower the survival probability of the corresponding observation objects are, vice versa. Refitting the 15 significant genes into the genetic model with leave one out cross validation, the prognostic indices *CVPI*_*gene*_ are obtained.

A bioinformatics analysis is performed on the 15 significant genes. Major functions related with KIRC are listed in Table A.4 in the Supplementary Materials. Some of these selected genes, such as CHRM4 and SLC17A4, involve in small molecule transport, tumour signal transduction and organism metabolism balance. Some are oncogenes or tumour suppressor genes, like TCN1 and SLC5A8. Some are supposed to be correlated with immune process, such as MTTP, OGN and INHBE. The 15 selected genes might play a substantial role in tumour formation, progression and metastasis, thus becoming candidate biomarkers and therapeutic targets for future research.

### Prognostic model construction

From the above analysis, we have the two cross-validated prognostic indices *CVPI*_*clin*_ and *CVPI*_*gene*_, which represent the efficient clinical and genetic information from the KIRC patients. To combine the two sources information, the two prognostic indices are fitted into the Super learner model as a new prognostic model. The fitted model is as follows:

$$h(t|CVPI)={h}_{0}(t)\,\exp \,(0.780\,\ast \,CVP{I}_{clin}+0.574\,\ast \,CVP{I}_{gene})$$

Similarly as *CVPI*_*clin*_ and *CVPI*_*gene*_, we define the internal content of exponential function of the Super learner model 0.780 * *CVPI*_*clin*_ + 0.574 * *CVPI*_*gene*_ as the combined indicator *CVPI*_*comb*_.

### Model performance comparison

**Figure 2:**
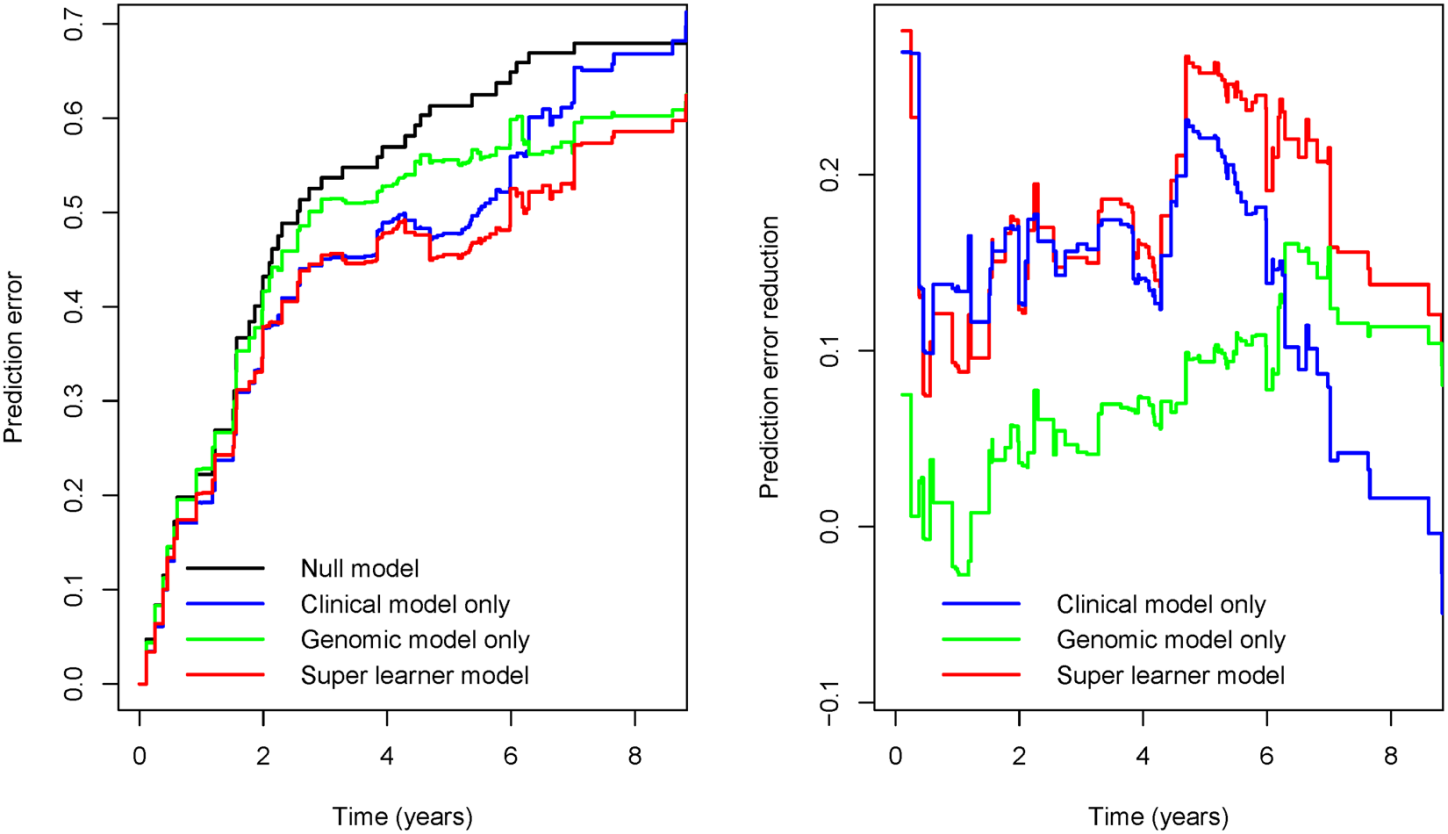
The prediction error curve (left) and the prediction error reduction curve (right) in full dataset.

We compare the performance of the clinical and genetic model with the Super learner model (Table 3 and Fig. 2). From the perspective of the model’s chi-square statistics (34.80 > 28.48 > 15.57), and the corresponding AUC value (0.835 > 0.79 1 > 0.737), it is observed that the Super learner model has the best performance for the full dataset. Then we split the full dataset into two parts according to the metastasis status. For the M0 dataset with no metastasis, genetic model and the Super learner model outperform the clinical model, while the Super learner model is slightly inferior to the genetic model. For the M1 dataset with confirmed distal metastasis, the Super learner model achieves the best performance, greatly outperform the clinical model. Besides, from the perspective of the model’s predictive performance, the prediction error curve (left) and the prediction error reduction curve (right) are obtained using Kullback-Leibler estimation. It can be seen from the left graph that the prediction error curves of the three models are below the prediction error curve of the zero model, and the error curve of the Super learner model is at the bottom, while in the right graph, the curve of the Super learner model is at the top, thus indicating that the three models all reduce the prediction error rate and the Super learner model achieves the most reduction.

**Table 3:** Regression coefficients and model performance of the clinical, genetic and Super learner model in three datasets.

		Clinical model	Genetic model	Super learner model
Full dataset	Clinical (*α*_1_)	0.900		0.780
	Gene (*α*_2_)		0.832	0.574
	Model (*χ*^2^)	28.48	15.57	34.80
	AUC	0.791	0.737	0.835
M0 dataset	Clinical (*α*_1_)	0.280		0.138
	Gene (*α*_2_)		0.541	0.519
	Model (*χ*^2^)	0.26	2.01	2.07
	AUC	0.594	0.612	0.607
M1 dataset	Clinical (*α*_1_)	0.464		0.886
	Gene(*α*_2_)		0.631	0.775
	Model (*χ*^2^)	1.14	4.61	7.78
	AUC	0.594	0.748	0.811

Therefore, the combination of clinical and genetic information has advantages over only a single source, especially suitable for patients with distal metastasis. As for patients with no metastasis, the Super learner model and genetic model both deserve consideration. Generally speaking, using the Super learner model to conduct dynamic prediction could provide more accurate results.

### Four representative patients’ dynamic prediction

Based on the original data, we simulate four patients. The selection method is as follows.

1. Sort *CVPI*_*clin*_ and *CVPI*_*gene*_ from small to large. Select the upper and lower quartile of *CVPI*_*clin*_ (0.785 and −0.714) and *CVPI*_*gene*_ (0.402 and −0.566) as the high and low risk division nodes.
2. Calculate the value of *CVPI*_*comb*_ of the above four different pairs of division nodes and treat 0.22, 0.41, 0.66 and 0.77 respectively as (A) the low-risk clinical and low-risk genetic patient, (B) the high-risk clinical and low-risk genetic patient, (C) the low-risk clinical and high-risk genetic patient and (D) the high-risk clinical and high-risk genetic patient. For the sake of convenience, the following explanation is replaced by the above letters.

Using the landmark method requires selecting different time nodes *t*_*LM*_ = s as new censorship criteria and *CVPI*_*comb*_ as an independent variable and then continually stacking to produce new datasets. Set the window width w = 5, the time range as 0–7 years, and the interval as 0.1 year. Considering the ipl* model with the landmark-dependent linear landmark interactions, we finally obtain the dynamic Super learner model:

$$\begin{array}{rcl}h(t|x,{t}_{LM}={\rm{s}},7) & = & {h}_{0}(t)\,\exp \,(0.5788\,s/7)-0.2867{(s/7)}^{2}\\  &  & +\,1.2051\,CVP{I}_{comb}-0.5703\,s/7\,CVP{I}_{comb})\end{array}$$

**Figure 3:**
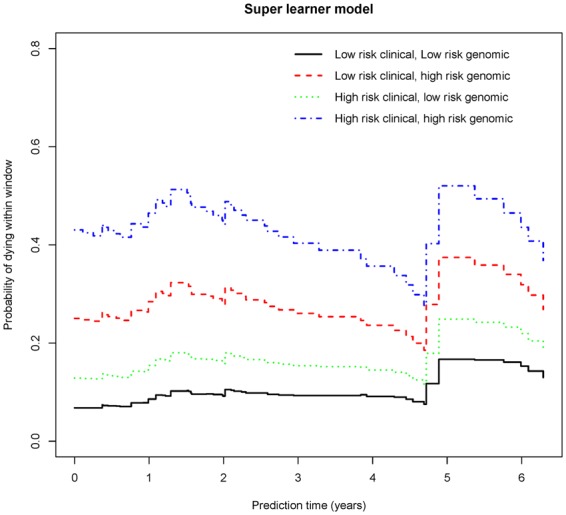
Four representative patients’ dynamic prognostic model results.

After obtaining the risk function, we can dynamically predict the mortality rate of the four patients selected above. Create the patient survival trend graph with the time (years) as the horizontal axis and the mortality rate within the fixed window width as the vertical axis. The graph is shown in Fig. 3.

It can be observed that the cumulative mortality rate of patient D is the highest (the peak reaches around 60%), followed by B, C and A (the peaks reaches around 20%), in decreasing order. The result indicates that high prognostic indices might be related with high mortality rates. The four mortality curves show the same trend. At 0–1.5 years, the mortality rate is on the slight rise, after that, the four curves all show a downward trend. It can be hypothesized that at a certain time, the doctor might conduct an efficient interventional treatment. It can also be hypothesized that the risk period of the disease is approximately 5 years since all the mortality probability curves show a rapidly upward trend around 5 years, and thus, patients who survive for longer than 5 years would present relatively good prognosis.

## Discussion

In this article, we aim to combine the clinical and genetic information into the Super learner model for more accurate prognosis and dynamic predictions, as well as some significant genes as candidate biomarkers or prognostic factors.

Through the general Cox proportional hazards model and LASSO-penalized Cox proportional hazards model, which are defined here as the clinical model and the genetic model, sufficient information is obtained in the two cross-validated prognostic indices. To achieve more accurate predictive performance, we combine the clinical and genetic information to build the Super learner model and introduce the time-varying effect into the combined model using the landmark method for real-time dynamic prediction.

The approach we used has obvious advantages over the typically preferred clinical model and genetic model for metastasis patients. Using the same type of information (clinical or genetic alone) has the one-sidedness that cannot be ignored. This problem can be effectively solved by combining two sources information into one prognostic model, such as the Super learner model. However, for patients with no metastasis, the Super learner model outperform the clinical model but a little inferior to the genetic model, indicating the value of the genetic model which is worth taking into consideration like the Super learner model in such situation. In addition, few reports have considered the updating information during the follow-up process, no matter whether the clinical model or the genetic model is static. Therefore, we try to introduce the time-varying effect into the prognostic model using the existing mature and classical landmark prediction method. In this way, the real-time tracking and prediction can be realized.

From the variable selection, information combination and dynamic prediction, the following conclusions can be drawn.

4 clinical indicators influencing the survival time of KIRC patients, including the tumour grade, tnm_m, necrosis and tumour size, are selected by the clinical model. As each of the four clinical indicators increases, the risk of death will also rise. Using the 4 clinical indicators, the UISS score and the SSIGN score as covariates separately, we compare the model performance of the three candidate models and finally determine the clinical model with SSIGN score.

15 genes are screened out from the genetic model. Among them, 10 genes are positively expressed, and the remaining are negative. That is, the expression change of the 10 genes may increase the risk, while the remaining 5 genes will reduce the risk. These selected genes involve in small molecule transport, tumour signal transduction, organism metabolism balance and immune process. They might play a substantial role in tumour formation, progression and metastasis, thus becoming candidate biomarkers and therapeutic targets for future research.

By combining the efficient clinical and genetic information from the two single source model, we successfully construct the Super learner prognostic model. From the chi-square test results, the AUC values and the predictive performance of the KL method, it can be concluded that the Super learner model is the best among the three models with a more accurate prognostic performance, thus proving the combination of clinical and genetic information has advantages over only a single source.

By introducing the time-varying effect into the Super learner model using the landmark method, it is found that if the patient’s two prognostic indices are both high (the two high-risk combinations), the mortality rate would rise to approximately 60%. However, if the two prognostic indices are both low (the two low-risk combinations), the mortality would fluctuate at approximately 20%. Therefore, effectively reducing the prognostic indices can achieve the purpose of controlling the mortality of patients. Apart from this, the risk period of the disease is approximately 5 years since all the mortality probability curves show a rapidly upward trend around 5 years, and thus, patients who survive for longer than 5 years would present relatively good prognosis.

## Conclusions

In general, combining clinical and genetic information into the dynamic prognostic model would enhance the predictive performance and yield more accurate results. On the one hand, the approach can help doctors realize the real-time monitoring of patients’ future mortality according to the continually updated clinical data and genetic data, thus finding the individual specific disease progression pattern or further refining the general characteristics of the specific disease. On the other hand, when the doctor conducts some kind of intervention (such as medication or surgery), updating the patient’s clinical data and genetic data once again to draw the dynamic prognosis graph can help doctors judge whether the treatment is effective for the patient and thus give the most efficient and personalized treatment to reduce the mortality, thereby improving the accuracy, fitness and success rate of treatments.

## Electronic supplementary material

Supplementary Materials

## Data Availability

The data used in this paper can be easily downloaded from the website.
