# Supplementary material for: Dynamic prognostic model for kidney renal clear cell carcinoma (KIRC) patients by combining clinical and genetic information
Source: Sci Rep. 2018 Dec 4;8:17613. doi: 10.1038/s41598-018-35981-5 (PMC6279814; doi:10.1038/s41598-018-35981-5)
Supplement: Supplementary file 1 — Supplementary Materials [file 41598_2018_35981_MOESM1_ESM.pdf]

Supplementary materials for Dynamic prognostic model for kidney renal clear cell carcinoma (KIRC) patients by combining clinical and genetic information

Huiling Zhao[1], Yuting Cao[1], Yue Wang[1], Liya Zhang[1], Chen Chen[1]Yaoyan Wang[1], Xiaofan Lu[1], Shengjie Liu[1], Fangrong Yan*

Research Center of Biostatistics and Computational Pharmacy, China pharmaceutical University, Nanjing 210009, P.R.China

Author names and affiliations:
Huiling zhao[1] zhaohuiling710@163.com
Yuting Cao[1] 352182595@qq.com
Yue Wang[1] 245578592@qq.com
Liya Zhang[1] 734497942@qq.com
Chen Chen[1] 940537206@qq.com
Yaoyan Wang[1] cpuwangyaoyan@163.com
Xiaofan Lu[1] 627922151@qq.com
Shengjie Liu[1] 626744398@qq.com
Research Center of Biostatistics and Computational Pharmacy, China pharmaceutical University, Nanjing 210009, P.R.China

Author for correspondence:
Fangrong Yan*
f.r.yan@163.com +86 18851607393
Research Center of Biostatistics and Computational Pharmacy, China pharmaceutical University, Nanjing 210009, P.R.China

Clinical and genetic model

The clinical model is defined as Eq.(A.1), in which Z represents the clinical variable and γ is the regression coefficient. Similarly, the genetic model is defined as Eq.(A.2), in which X represents gene expression matrix, β is the regression coefficients. Difference is that LASSO method is introduced into Eq.(A.2) to reduce the dimensions, where β is under the penalty constraint.

$$h(t \mid Z) = h_0(\mathrm{t})\exp(\mathrm{Z}^T \gamma) \qquad \text{Eq.(A.1)}$$

$$h(t \mid X) = h_0(t)\exp(\mathrm{X}^T \beta)$$

$$\hat{\beta} = \operatorname{argmax} l(\beta), \text{ subject to } \sum_{j=1}^{\rho} \left|\beta_j\right| \le \lambda \qquad \text{Eq.(A.2)}$$

$$l(\beta) = \log L(\beta),\ L(\beta) = \frac{\exp(\beta^T X_i)}{\sum_{j \in R_i} \exp(\beta^T X_j)}$$

$$\hat{S}_{CV,i}(t) = \hat{\mathrm{S}}_{(-\mathrm{i})}(t \mid x_i) = \exp(-\mathrm{H}_{0,(-i)}(t) \cdot \exp(x_i^T \hat{\beta}_{(-i)}))$$

$$CVPI_i = \ln(-\ln(\hat{\mathrm{S}}_{CV,i}(t_0))) \qquad \text{Eq.(A.3)}$$

After the first screening step from logarithmic rank test and Kaplan-Meier (KM) estimation, significant clinical variables which have differences on the survival between the cancer group and the control group are fitted into the clinical model for the second screening. The remaining clinical indicators are re-fitted into Eq.(A.1) and the cross-validated prognostic indices $CVPI_{clin}$ are obtained from Eq.(A.3). For UISS and SSIGN model, all are the same except the clinical indicators. Comparing the performance of the three candidate models with selected indicators, UISS and SSIGN as variables, the best model is selected as the clinical model. Similarly, after the first screening from the LASSO-based Cox model, significant genes are re-fitted into Eq.(A.2), the cross-validated prognostic indices $CVPI_{gene}$ are obtained in the same way. Two sources prognostic information is then used to fit the Super learner model.

Super learner model

The specific form of the Super learner model Eq.(A.4) is as follows, in which α_1 and α_2 are the corresponding regression coefficients.

$$h(t \mid CVPI) = h_0(t)\exp(\alpha_1 CVPI_{clin} + \alpha_2 CVPI_{gene}) \qquad \text{Eq.(A.4)}$$

Through this approach, the patients' clinical and genetic information are combined into a single survival model, and the following dynamic prediction is performed on it.

Landmark dynamic prediction

To some extent, the patients' data is dynamically updated, so the prognostic model should be constantly updated based on the latest data in order to avoid inaccuracies. To achieve this goal, based on the super learner model, we establish multiple time intercept points $\{s_1, s_2, \cdots, s_l\}$ and create a prediction data set for each $t_{LM} = s_l$ by truncation and administrative censoring. After that, we stack all those datasets into a single "super prediction dataset". In the large super prediction dataset, the subset corresponding to a given prediction time $t_{LM} = s_l$ is labelled "strata". Passing from one stratum to the next one corresponds to sliding the time window over the time range. With the window width defined as w, the landmark prediction model Eq.(A.5) is presented as follows:

$$h(t \mid x, t_{LM} = s, w) = h_0(t \mid s, w)\exp(x^T \beta_{LM}(s)), s \le t \le s + w \qquad \text{Eq.(A.5)}$$

Let the regression coefficients β_{LM} depend on $t_{LM} = s$ in a smooth way and model that in Eq.(A.6) in a linear way.

$$\beta_{LM}(s) = \sum_{j=1}^{m} \gamma_j f_j(s) \qquad \text{Eq.(A.6)}$$

The baseline hazard function depends on s via the smooth function $\widehat{\beta}_{LM}(s)$. This smoothness could be modelled in Eq.(A.7) directly by letting

$$h_0(t \mid s, w) = h_0(t)\exp(\theta(s)), \theta(s) = \sum_{j=1}^{k} \eta_j g_j(s) \qquad \text{Eq.(A.7)}$$

Through the above transformation, the landmark prediction model can be fitted by applying a Cox model without stratification with the main effects for the stratum variable s modelled by $\theta(s)$ and the interaction of s with the covariates modelled by $\beta(s)$. In this article, we define $\theta(s)$ and $\beta(s)$ as Eq.(A.8) and Eq.(A.9).

$$\theta(s) = \eta_1(s/7) + \eta_2(s/7)^2 \qquad \text{Eq.(A.8)}$$

$$f(s) = \gamma_1 CVPI_{comb} + \gamma_2 \frac{s}{7} CVPI_{comb} \qquad \text{Eq.(A.9)}$$

The result leads to a pseudo partial log-likelihood model ipl^*, which is given by Eq.(A.10)

$$ipl^*(\gamma, \eta) = \sum_{i=1}^{n} d_i \ln\left(\frac{\sum_{\{s \mid s \le t_i \le t+w\}} \exp(x_i^T \beta_{LM}(s \mid \gamma) + \theta(s \mid \eta))}{\sum_{\{s \mid s \le t_i \le t+w\}} \sum_{j \in R(t_i)} \exp(x_j^T \beta_{LM}(s \mid \gamma) + \theta(s \mid \eta))}\right) \qquad \text{Eq.(A.10)}$$

The corresponding estimate of the baseline hazard is given by Eq.(A.11)

$$\widehat{h}_0(t_i) = \frac{\#\{s \mid s \le t_i \le s+w\}}{\sum_{\{s \mid s \le t_i \le t+w\}} \sum_{j \in R(t_i)} \exp(x_j^T \beta_{LM}(s \mid \gamma) + \theta(s \mid \eta))} \qquad \text{Eq.(A.11)}$$

Predictions in the $ipl*$ model are obtained for all $s \in \{s_1, s_l\}$ by Eq.(A.12)

$$\widehat{H}(s+w \mid x, t_{LM} = s) = \exp(x^T \widehat{\beta}_{LM}(s) + \widehat{\theta}(s))(\widehat{H}_0^*(s+w) - \widehat{H}_0^*(s-)) \qquad \text{Eq.(A.12)}$$

By using the landmark $ipl*$ integral partial logarithmic likelihood model that contains both the effects of the stratified variables and the interaction effects of the stratified variables and covariates to introduce the time-varying effect, the accurate dynamic prediction of a patient's future survival rate can be realized.

Table A.1 Clinical indicators summary table

Variable name	Content	Sample size
Tumor grade	G1/2	62
	G3	60
	G4	30
Laterality	right	81
	left	71
Gender	female	60
	male	92
Tnm_m	M0	111
	M1	41
Tnm_n	N0/Nx	145
	N1	7
Tnm_t	T1	63
	T2	21
	T3	66
	T4	2
ESCO_group	0	103
	1	41
	2	7
	3	1
Tumor_size	<=7cm	91
	>7cm	61
Nuclei_percent	<=80%	64
	>80%	88
Necrosis	yes	65
	no	87
Diag_age	<=60	83
	>60	69
UISS	1	25
	2	79
	3	11
	4	26
	5	11
SSIGN	0	15
	1	4
	2	21

3	21
4	15
5	13
6	5
7	15
8	6
9	14
10	1
11	11
13	10
15	1

Table A.2 univariate logarithmic rank test results

Clinical indicator	P value	Clinical indicator	P value
Tumour grade	<0.001***	Diag_age	0.6
Tumour laterality	0.04*	Gender	0.6
Tnm_m	<0.001***	UISS score	<0.001***
Tnm_n	0.01*	Necrosis	0.04*
Tnm_t	0.002**	Nuclei_percent	0.06
Tumor_size	0.004**	SSIGN score	<0.001***

p<0.05(*), <0.01(**)， <0.001(***)

Table A.3 Genes screened out from the genetic model

Number.	Gene name	Parameter
1	SLC17A4	-0.038
2	INHBE	0.041
3	IGFN1	0.084
4	L1CAM	0.022
5	ZIC2	0.075
6	TCN1	0.052
7	KCND2	0.035
8	CHRM4	0.016
9	MTTP	0.044
10	SLC5A8	-0.003
11	HBG1	-0.051
12	OGN	-0.064
13	IGF2BP2	0.015
14	CNTNAP5	-0.035
15	OR12D2	0.014

Table A.4 selected gene functions

gene	function	references
SLC17A4	anion transporter family member, NPT homologue, possible involvement in the control of phosphate excretion from the kidney	Togawa N (2012) [1]
INHBE	a growth factor belongs to TGF-β family, generate an inhibin βE subunit, substantial roles in endocrine-responsive tumours, a potential immune target	Bergauer F (2009) [2]
IGFN1	a complex locus that codes for multiple splicing variants of Immunoglobulin- and Fibronectin-like domain containing proteins	Li X (2017) [3]
ZIC2	a significant correlation between renal/urinary defects and mutations of ZIC2.	Mercier S (2011) [4]
TCN1	an important oncogene for various cancers of the breast, lung ,stomach, prostate, etc.	Lee YY (2017) [5]<br>Claerhout S (2011) [6]<br>Collin SM (2011) [7]
KCND2	Voltage-gated K+ channel family member, a hub gene and a valuable prognostic biomarker in the complex metabolic network of various cancers	Langford DJ (2015) [8]<br>Wei H (2018) [9]<br>Chen J (2017) [10]
CHRM4	muscarinic cholinergic receptors family member, involve in some cancer signalling pathways	Noh KM (2012) [11]
L1CAM	markers and prognostic factors of many human cancers, confers bad prognosis and augments cell motility, invasion and metastasis, might be promising tools for cancer therapy	Chen SC (2017) [12]<br>Doberstein K (2015) [13]
MTTP	regulate the CD1 family, a unique regulator of human metabolic and immune pathways	Hussain MM (2012) [14]
SLC5A8	a potential tumour suppressor gene in colon, thyroid, stomach, kidney, brain, etc.	Zhang Y (2010) [15]<br>Ganapathy V (2005 [16]<br>Ganapathy V (2008) [17]
HBG1	a γ-globin gene affect levels of fetal hemoglobin	Acuto S (1996) [18]
OGN	a potential immune target, candidate markers for some cancers	Hu X (2018) [19]<br>Liu L (2018) [20]<br>Hu X (2018) [21]

IGF2BP2	dysregulation is associated with carcinogenesis, a candidate biomarker and target for cancer	Cao J (2018) [22]
CNTNAP5	Circulating tumor cells (CTC) associated CAN	Chiu CG (2014) [23]
OR12D2	interacting with products of genes of the major histocompatibility complex (MHC)	da Silva JS (2013) [24]

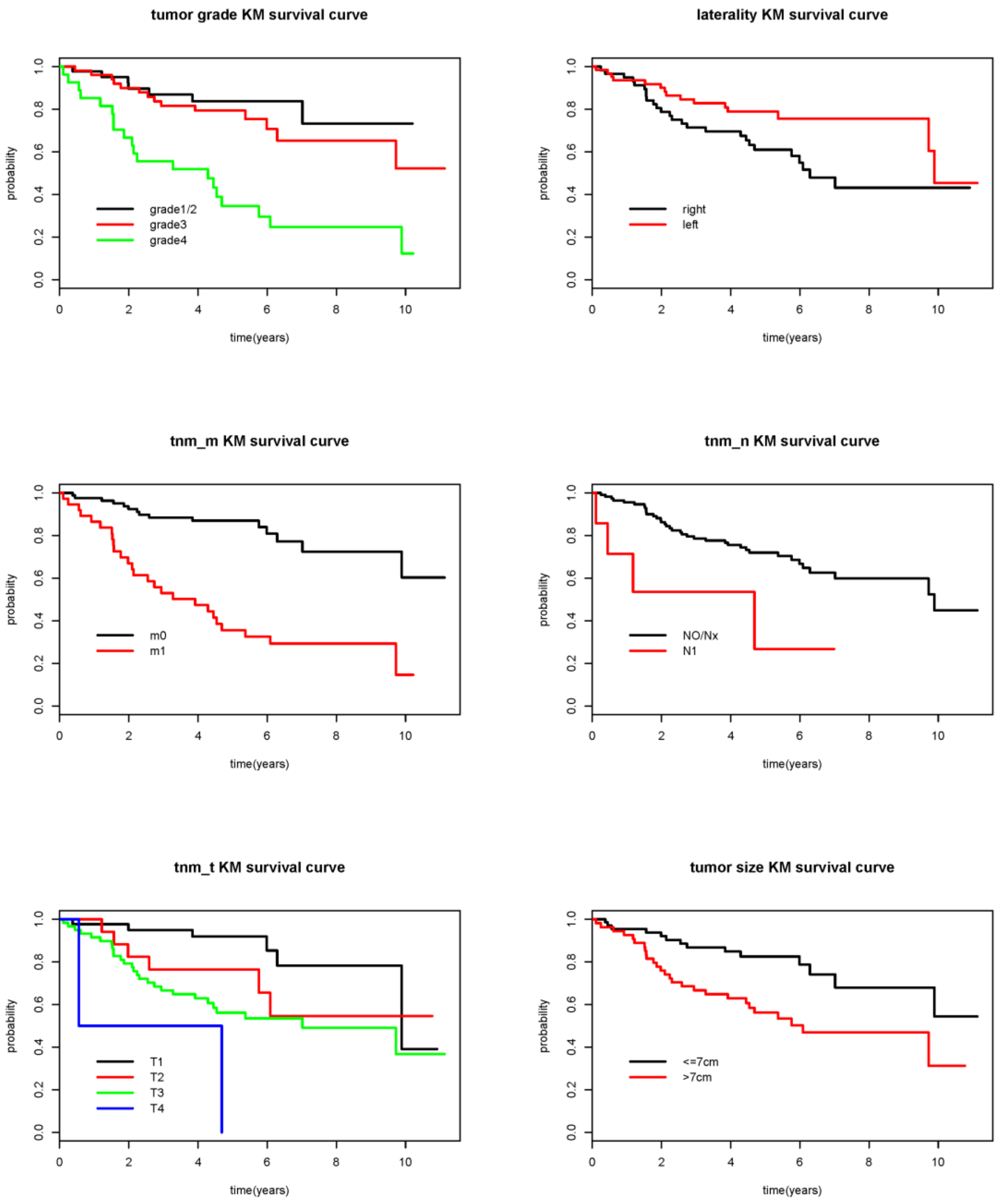

Figure A.1 KM curves of the tumour grade, tumour laterality, tnm_m, tnm_n, tnm_t and tumour size

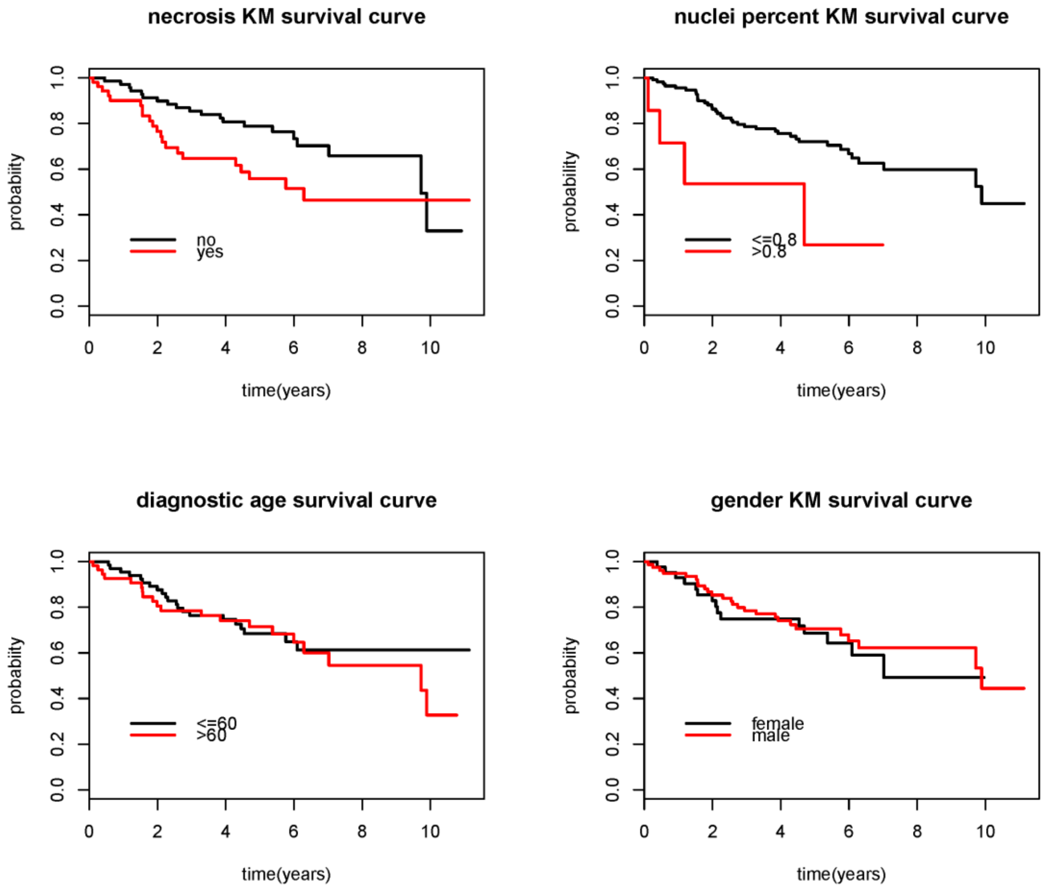

Figure A.2 KM curves of the necrosis, nuclei_percent, diag_age and gender

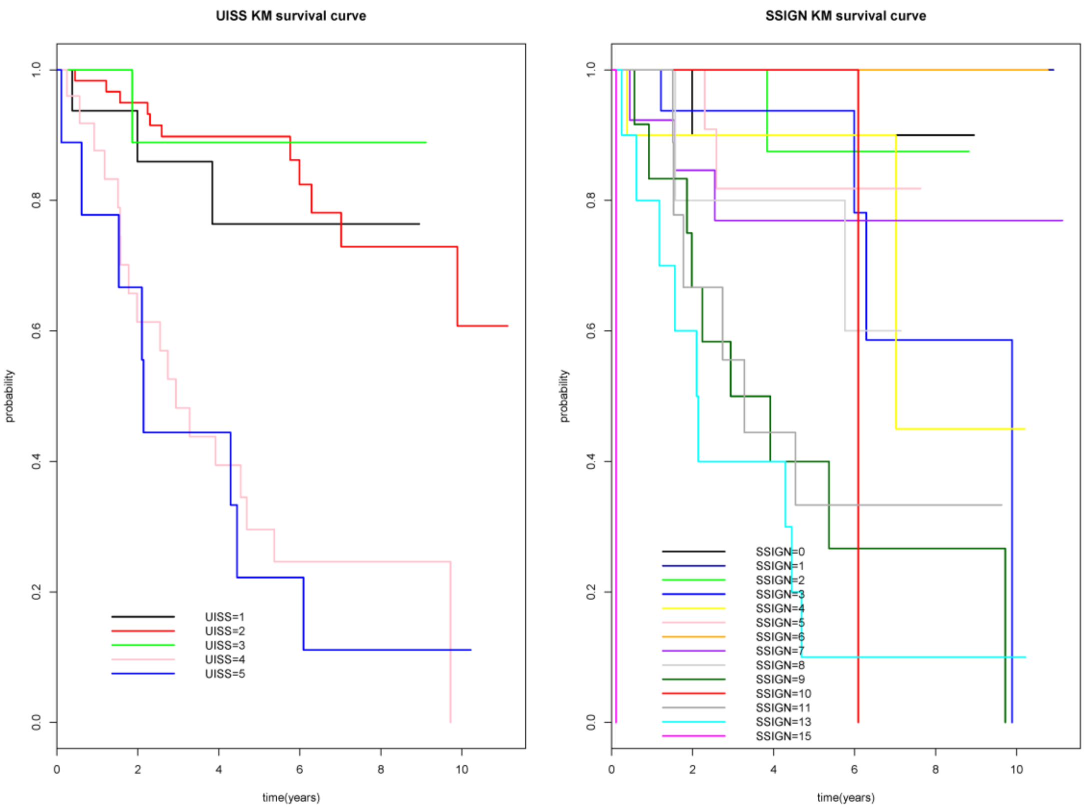

Figure A.3 KM curves of the UISS score and SSIGN score

References

[1] Togawa, N. et al. A Na+-phosphate cotransporter homologue (SLC17A4 protein) is an intestinal organic anion exporter. J. American Journal of Physiology-Cell Physiology. **302,** 1652-1660 (2012).

[2] Sugiyama, M. et al. Inhibin βE (INHBE) is a possible insulin resistance-associated hepatokine identified by comprehensive gene expression analysis in human liver biopsy samples. J. PloS one. **13,** e0194798 (2018).

[3] Li, X. et al. IGFN1_v1 is required for myoblast fusion and differentiation. J. PloS one. **12,** e0180217 (2017).

[4] Mercier, S. et al. New findings for phenotype–genotype correlations in a large European series of holoprosencephaly cases. J. Journal of medical genetics. **48,** 752-760 (2011).

[5] Lee, Y. Y. et al. Overexpression of transcobalamin 1 is an independent negative prognosticator in rectal cancers receiving concurrent chemoradiotherapy. J. Journal of Cancer. **8,** 1330 (2017).

[6] Claerhout, S. et al. Gene expression signature analysis identifies vorinostat as a candidate therapy for gastric cancer. J. PloS one. **6,** e24662 (2011).

[7] Collin, S. M. et al. The causal roles of vitamin B12 and transcobalamin in prostate cancer: can Mendelian randomization analysis provide definitive answers. J.

International journal of molecular epidemiology and genetics. **2,** 316 (2011).
[8] Langford, D. J. et al. Variations in potassium channel genes are associated with distinct trajectories of persistent breast pain after breast cancer surgery. J. Pain. **156,** 371-380 (2015).
[9] Wei, H., Li, Y., Du, Y. W. & Ma, J. KCND2 upregulation might be an independent indicator of poor survival in gastric cancer. J. Future Oncology. Published Online at https://doi.org/10.2217/fon-2018-0418 (2018).
[10] Chen, J. et al. Silencing TAK1 alters gene expression signatures in bladder cancer cells. J. Oncology letters. **13,** 2975-2981 (2017).
[11] Noh, K. M. et al. Repressor element-1 silencing transcription factor (REST)-dependent epigenetic remodeling is critical to ischemia-induced neuronal death. J. Proc Natl Acad Sci USA. **109,** 5928 -5929 (2012).
[12] Chen, S. C. et al. Systematic Analysis of Transcriptomic Profile of Renal Cell Carcinoma under Long-Term Hypoxia Using Next-Generation Sequencing and Bioinformatics. J. International journal of molecular sciences. **18,** 2657 (2017).
[13] Doberstein, K. et al. Antibody therapy to human L1CAM in a transgenic mouse model blocks local tumor growth but induces EMT. J. International journal of cancer. **136,** 326-339 (2015).
[14] Hussain, M. M. et al. Multiple functions of microsomal triglyceride transfer protein. J. Nutrition & metabolism. **9,** 14 (2012).
[15] Zhang, Y. et al. Activin A induces SLC5A8 expression through the Smad3 signaling pathway in human colon cancer RKO cells. J. The international journal of biochemistry & cell biology. **42,** 1964-1972 (2010).
[16] Ganapathy, V. et al. Biological functions of SLC5A8, a candidate tumour suppressor. J. Biochemical Society Transactions. **33,** 237 -240 (2005).
[17] Ganapathy, V. et al. Sodium-coupled monocarboxylate transporters in normal tissues and in cancer. J. The AAPS journal. **10,** 193-199 (2018).
[18] Acuto, S. et al. An element upstream from the human δ-globin-encoding gene specifically enhances β-globin reporter gene expression in murine erythroleukemia cells. J. Gene. **168,** 237-241 (1996).
[19] Hu, X. et al. Osteoglycin-induced VEGF Inhibition Enhances T Lymphocytes Infiltrating in Colorectal Cancer. J. EBioMedicine. **34,** 35-45 (2018).
[20] Liu, L. et al. Identification of Key Candidate Genes and Pathways in Endometrial Cancer by Integrated Bioinformatical Analysis. J. Asian Pacific journal of cancer prevention: APJCP. **19,** 969-975 (2018).
[21] Hu, X. et al. Osteoglycin (OGN) reverses epithelial to mesenchymal transition and invasiveness in colorectal cancer via EGFR/Akt pathway. J. Journal of Experimental & Clinical Cancer Research. **37,** 37-41 (2018).
[22] Cao, J., Mu, Q., Huang, H. The Roles of Insulin-Like Growth Factor 2

mRNA-Binding Protein 2 in Cancer and Cancer Stem Cells. J. Stem Cells International. **2018,** 1-15 (2018).
[23] Chiu, C. G. et al. Genome-wide characterization of circulating tumor cells identifies novel prognostic genomic alterations in systemic melanoma metastasis. J. Clinical Chemistry. **60,** 873-885 (2014).
[24] Da, S. J. et al. Absence of strong linkage disequilibrium between odorant receptor alleles and the major histocompatibility complex. J. Human immunology. **74,** 1619-1623 (2013).
